# Development and Validation of the Agency in Contraceptive Decisions Scale in Uganda and Nigeria

**DOI:** 10.1111/sifp.70033

**Published:** 2025-09-22

**Authors:** Sneha Challa, Ushma D. Upadhyay, Ronald Wasswa, Sylvia Nanono, Ivan Idiodi, Chioma Okoli, Phoebe Alitubeera, Dinah Amongin, Ayobambo Jegede, Aminat Tijani, Catherine Birabwa, Lynn Atuyambe, Shakede Dimowo, Grace Nmadu, Christine Dehlendorf, Elizabeth Omoluabi, Peter Waiswa, Kelsey Holt

## Abstract

To fill a gap in measures of empowered contraceptive decision‐making, we developed the Agency in Contraceptive Decisions Scale in Uganda and Nigeria. We developed an item pool drawing on the previously published Contraceptive Agency framework. We refined items through cognitive interviews (*N* = 80) and expert feedback and piloted a reduced item pool via surveys (*N* = 3002). Exploratory factor analysis using a random half of the sample suggested a 15‐item scale (Cronbach's α = 0.8) including four subscales: (1) Beliefs about Rights and Perceived Decision‐making Control (α = 0.8), (2) Decision‐making Self‐efficacy (α = 0.8), (3) Knowledge Aligned with Preferences (α = 0.8), and (4) Control over Use or Non‐use (α = 0.8). Confirmatory factor analysis with the other half of the sample supported this solution. Agency in Contraceptive Decisions Scale scores were significantly associated with scores on the contraceptive existence of choice and contraceptive exercise of choice subscales of the Women's and Girls Empowerment in Sexual and Reproductive Health Index, supporting construct validity. The 15‐item Agency in Contraceptive Decisions Scale and individual subscales are valid and reliable for use in Nigeria and Uganda. This measure offers an innovative alternative for gauging the success of contraceptive programs and policies in advancing the right to empowered choices.

## BACKGROUND

Despite widespread concern that a singular focus on use of modern contraception does not reflect a human rights‐based approach to contraception (Senderowicz and Maloney 2022; Dehlendorf et al. 2018; Potter et al. 2019; Speizer, Bremner, and Farid 2022; Holt et al. 2023), global contraceptive programming still prioritizes contraceptive use‐focused measures as primary metrics of success. The ability to make contraceptive choices is a basic human right that applies regardless of what these choices are (World Health Organization 2014). Thus, part of the call to align measurement frameworks and prioritized outcomes with rights‐based principles has been to highlight the need for measures of choice that focus on whether the decision has been empowered, rather than what the decision was. These measures should acknowledge the range and diversity of legitimate decisions people make around contraceptive use or nonuse (Holt et al. 2024, 2023; Senderowicz 2020; Moreau et al. 2020).

Existing measures of contraceptive decision‐making are limited in several ways (Holt et al. 2024): first, measures tend to equate (overt) contraceptive use with empowerment. For example, items ask about whether individuals could convince a partner to use contraception, but not about whether they could convince a partner *not to* or whether they have the confidence to use covertly if needed. Second, few measures capture critical reflection, a concept from critical consciousness theory that refers to awareness of systemic inequity (Friere 2020), widely recognized to be a critical internal process related to the formation of values‐based preferences (Donald et al. 2020; Edmeades et al. 2018). Third, existing measures rely heavily on items about partner influence at the expense of broader items about other potential sources of social influence, such as family members or healthcare providers. Further, items about others’ role in decision‐making tend to equate partners having an outsized role with a lack of empowerment, without acknowledging that in many cases women may highly value partners’ opinions; one notable exception is the reproductive decision‐making agency measure (Hinson et al. 2019). Finally, existing measures are focused on women, missing an opportunity to be able to gauge men's and gender nonbinary people's decision‐making agency related to the methods they can use.

We published the Contraceptive Agency Framework (Holt et al. 2024) in response to this need for better measures of empowerment in contraceptive decision‐making, in alignment with other global efforts increasingly prioritizing a focus on agency (Hinson et al. 2019; Harper et al. 2023; Agency for All Project 2023) and building on conceptual and measurement innovations in sexual and reproductive health more broadly (Edmeades et al. 2018; Upadhyay et al. 2014; Karp et al. 2020; Moreau et al. 2020). The theoretically informed framework defines contraceptive agency as “the ability of an individual to make and act on decisions related to whether to do something to avoid or delay pregnancy and what, if anything, to do when they are not actively trying to become pregnant” (Holt et al. 2024). It specifies eight measurable constructs that comprise contraceptive agency.

In this paper, we describe our process of developing a gender neutral measure to capture the constructs outlined in the Contraceptive Agency Framework. This study took place in the context of the Innovations for Choice and Autonomy (ICAN) project; ICAN's main aim was to understand how self‐injection of the contraceptive *subcutaneous depot medroxyprogesterone acetate* (DMPA‐SC) can be implemented and scaled in a way that best supports women's agency and autonomy. An additional aim of ICAN was to develop and validate new rights‐based measures that could be used to evaluate the success of self‐injection and other contraceptive programming.

### Study Setting

Our measure development process for the Agency in Contraceptive Decisions Scale took place in two of the four ICAN countries: Uganda and Nigeria. These two varied contexts provided the opportunity to develop and test the new measure in populations with diverse needs, preferences, and experiences. Uganda was chosen as a study country because it has one of the most mature DMPA‐SC markets with large investments in research and programming focused on self‐injection implementation (Burke et al. 2014; Cover et al. 2017, 2018; Morozoff et al. 2022; PATH 2018). Additionally, it is characterized by a relatively high contraceptive prevalence of 41 percent among married women (Performance Monitoring for Action (PMA) 2022). In comparison, there is a lower contraceptive prevalence in Nigeria of 12 percent among married women (Adedini, Chizomam Ntoimo, and Alex‐Ojei 2023). In the Nigerian context, contraception is highly stigmatized (Sinai et al. 2020; Gueye et al. 2015; Fasanu et al. 2025; Ezenwaka et al. 2020), but the regulatory environment has made key advancements to support the provision of DMPA‐SC for self‐injection (Nigeria Federal Ministry of Health 2014; FMOH 2018; Akinyemi et al. 2022).

## METHODS

### Overview

**FIGURE 1:**
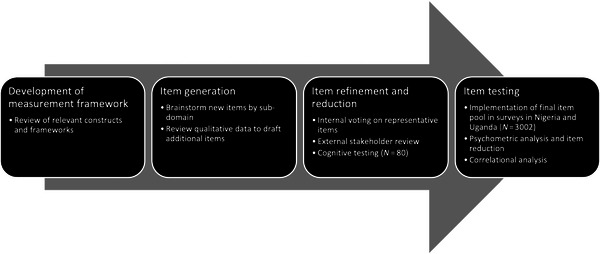
Overview of scale development and validation process with participation from American, Nigerian, and Ugandan teams

We developed and validated the Agency in Contraceptive Decisions Scale in Nigeria (Enugu and Plateau States) and Uganda (Oyam, Mayuge, Iganga, Kole, and Lira Districts) through a multistage process including four phases: (1) development of measurement framework, (2) item generation, (3) item refinement, and (4) item testing and reduction (Figure 1).

### Measurement Framework Development

**FIGURE 2:**
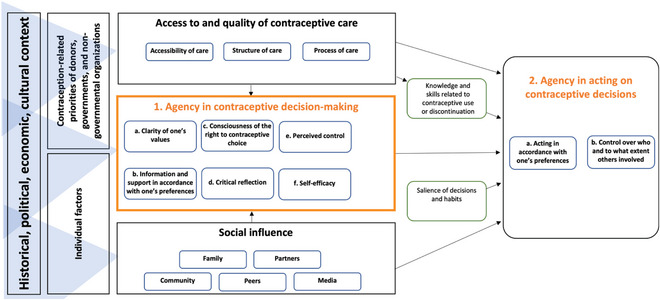
Contraceptive agency measurement framework NOTE: Figure 2 is a reprint from Holt K, Challa S, Alitubeera P, et al. Conceptualizing Contraceptive Agency: A Critical Step to Enable Human Rights‐Based Family Planning Programs and Measurement. *Global Health: Science and Practice* 2024;12(1):e2300299. https://doi.org/10.9745/GHSP‐D‐23‐00299

The process of developing our measurement framework, the Contraceptive Agency Framework (Figure 2), is described elsewhere (Holt et al. 2024). Briefly, the framework was informed by interdisciplinary theories of health behavior (specifically, the Integrated Behavior Model) (Montaño and Kasprzyk 2015), critical consciousness (Watts, Diemer, and Voigh 2011), and agency (Donald et al. 2020; Sato, Sayanagi, and Yanagihara 2022). The framework comprehensively covers constructs from the two domains of contraceptive agency: (1) agency in *decision‐making* related to avoiding or delaying pregnancy and (2) agency in *acting on decisions* related to avoiding or delaying pregnancy. Domain 1 constructs include clarity of values, information, and support, consciousness of rights, critical reflection, perceived control, and self‐efficacy, while Domain 2 constructs include acting in accordance with preferences and control over others’ involvement (see Figure 2).

### Item Generation

In this phase, we generated a new set of items representing the two domains and eight constructs from our measurement framework. We generated novel items through group brainstorming sessions attended by members of American, Nigerian, and Ugandan teams. Meetings included study Co‐PIs and staff from diverse educational, disciplinary, cultural, and language backgrounds to ensure a variety of perspectives were represented. Two resources were referred to by group members as inspiration during item generation. First, we collated a database of items abstracted from related measures (Upadhyay et al. 2014; Vedam et al. 2017; Afulani et al. 2017; Klima et al. 2015; Farrell, Simpson, and Rothman 2015; Borghei et al. 2015; Quinn‐Nilas et al. 2016; Pulerwitz, Gortmaker, and DeJong 2000; Ewerling et al. 2017; Hameed et al. 2014; Moonzwe Davis et al. 2014), organized by measurement framework construct. No items were borrowed verbatim from existing measures, but phrasing and content provided inspiration for the brainstorming process. Second, we referred to analytic memos from a four‐country, grounded theory qualitative study that was being simultaneously conducted as part of the ICAN project to characterize contraceptive decision‐making (Suchman, Himes, et al. 2025). Members of our team involved in analysis for this study referred to memos from Uganda and Nigeria to see how participants themselves were describing constructs represented in our measurement framework. This initial process resulted in 80 items.

### Item Refinement and Reduction

#### Initial Refinement

We reduced the item pool by programming the items into a Qualtrics survey that all authors used to vote on the most highly regarded items. When voting, authors were asked to focus on universal applicability, content validity, clarity, representation of constructs, and uniqueness. By tabulating results from the Qualtrics survey, we were able to see which items received the least votes to consider for removal. We shared the results back with the full team to elicit any input on the breadth and depth of construct coverage before we made final decisions on which items to retain/remove. We aimed for each of the eight constructs in the measurement framework to be represented by at least two items. We shared the resulting item pool with six family planning, gender equity, and human rights experts from the United States and sub‐Saharan Africa (external to our team) who provided comprehensive feedback. Edits were made based on their feedback, and the item pool at the end of this stage consisted of 68 items and two possible response sets. These items were translated by Nigerian and Ugandan research team members fluent in local languages.

#### Cognitive Interview Data Collection

We conducted cognitive testing (Uganda: July–September 2020; Nigeria: April–June 2021) to ensure items were universally comprehended and interpreted by participants. Cognitive interviews were conducted with a convenience sample of 80 participants (*n* = 40 per country), including *n* = 60 women (*n* = 30 per country) and *n* = 20 men (*n* = 10 per country). Recruitment was led by our Ugandan and Nigerian teams, supported by community health workers who helped ensure diversity across the study sites, age groups (15–19 vs. 20+), and contraceptive use statuses (users vs. nonusers). At a time and place of participants’ choosing, the research assistants (RAs) obtained informed consent and conducted interviews that lasted approximately one hour. To ensure that interviews were an appropriate length and limited participant fatigue, the item pool was randomly halved for each participant.

Cognitive interviews were conducted by trained RAs fluent in local languages. All teams worked together to ensure interviewer training materials were comprehensive and culturally relevant. Training lasted five days and included an overview of the Contraceptive Agency Framework and in‐depth sessions on cognitive interview procedures, including practice and role‐play. RAs were provided with an interview guide (see Online Appendix 1 for the cognitive interview instrument) that contained the item pool and probes to elicit participants’ feedback on the difficulty and comprehension of items.

#### Cognitive Interview Analysis

**FIGURE 3:**
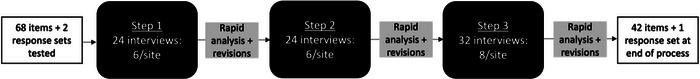
Cognitive interview protocol

To analyze cognitive interview data, we employed a structured, iterative item revision process (Figure 3) such that we conducted a subset of interviews, then paused to conduct rapid data review/analysis. We collected three types of data: (1) audio recordings of the interviews, (2) quantitative responses to understand variability in responses, and (3) voice memos for in‐depth interviewer insights post‐interview, all of which were all assessed to determine modifications to the item pool. Interviews and post‐interview memos were transcribed during pauses in data collection and reviewed to determine item‐level modifications. Transcripts were reviewed by a subset of American, Nigerian, and Ugandan study team members who recorded notes on inconsistent interpretations, misinterpretations, inappropriate phrasing, and suggestions for modification in a comprehensive spreadsheet. This spreadsheet was then reviewed by American team members who met to decide on modifications to implement for subsequent rounds of interviews. Then, the modified item pool was tested in the next step with a new set of participants. This process was repeated three times.

When all interviews were complete, the full study team again completed Qualtrics surveys to vote on the items that were best/most uniformly understood by participants, and which best represented our definition of contraceptive agency. Again, we shared the results with the full team to elicit any input on the breadth and depth of construct coverage before we made final decisions on which items to retain/remove. The final item pool included 42 items.

### Item Testing and Reduction

#### Study Design and Data Collection

The item pool for the Agency in Contraceptive Decisions Scale was tested in ICAN surveys in Uganda (November 2022–April 2023) and Nigeria (February 2023). In Nigeria, the interviewer‐administered survey was fielded as part of a mixed‐methods evaluation of efforts to increase access to self‐injectable contraception in public and private sectors of the healthcare system. The sampling frame included facilities that had participated in provider training for offering self‐injectable contraception. Our goal was to recruit at least 500 contraceptive users, but the sample size was primarily driven by client volume at participating facilities. Thus, data were collected from a facility‐based sample of sexually active women of reproductive age who were using contraception (*N* = 580) from two local government areas in Enugu and Plateau States. Nigerian authors trained RAs (female and fluent in local languages) to carry out recruitment and data collection procedures with women included in client registers at public health facilities. All clients on the registers who had accessed self‐injectable contraception in the past four months were invited, with the goal of recruiting at least 300 users of this method. To diversify the sample, RAs contacted users of other methods (aiming to recruit at least 200), beginning with the most recent visits on the registers until the target sample was achieved. Contact was established over the phone, and those who agreed to participate dictated a time and place for their in‐person survey. After obtaining verbal consent, RAs administered the survey, which took approximately 45 minutes. Additionally, RAs took advantage of immunization days at the facilities to conduct some in‐person recruitment and survey administration. After completion of the survey, participants were compensated approximately US $6.

In Uganda, the survey was fielded as part of a study aiming to understand the relationship between selecting self‐injectable contraception and contraceptive agency. As a result, our focus was on recruiting women who had initiated a new contraceptive method in the two weeks prior to survey administration. For the parent study, our power calculation was based on our primary research question regarding the relationship between self‐injectable contraception and contraceptive agency. The calculations indicated we needed to achieve a sample of *n* = 300 new self‐injectable contraceptive users and *n* = 1700 new users of other methods. We also aimed to recruit a sample of *n* = 400 nonusers to ensure the scale was valid in this sample. Therefore, all participants were either “new” users of a contraceptive method or contraceptive nonusers. To achieve the planned sample size in Uganda, recruitment was conducted in five districts: Oyam, Mayuge, Iganga, Kole, and Lira. We recruited sexually active women of reproductive age (*N* = 2422). Participants were recruited by trained female RAs fluent in local languages using client registers, referrals from providers and local community health workers, word‐of‐mouth, and active recruitment by study staff at drug shops/pharmacies/health facilities. Surveys took 60–90 minutes to administer by RAs after obtaining informed consent, and participants were compensated approximately US $8.

#### Measures

In Nigeria, surveys included items related to women's experiences with contraceptive services, including the quality of contraceptive counseling, as well as their decision‐making around method choice, method satisfaction, and our item pool. In Uganda, surveys included items on contraceptive use history, contraception‐seeking experiences, social norms related to contraception, contraceptive decision‐making, and our item pool.

*Contraceptive Agency Item Pool*. The 42‐item pool was administered with responses on a four‐point Likert scale using a two‐step process derived from the cognitive interview findings. Participants were first asked to respond “yes” or “no.” If participants responded “yes,” they were then asked to clarify whether it was “yes” or “strongly yes.” Similarly, if participants responded “no,” they were asked to clarify whether it was “no” or “strongly no.” Participants were also given a “no response” option, which was treated as missing in analyses.

*Participant Demographics*. We collected basic information about participants’ age, education, marital status, religion, and number of children. Age and number of children were captured continuously, while education included five categories (none, less than primary, primary, secondary, and college/university), marital status included three categories (married/partnered, not currently married/partnered, never married), and religion included three categories (Christianity, Islam, Other).

*Sexual and Reproductive Empowerment*. We included two subscales of the Women's and Girls’ Empowerment in Sexual and Reproductive Health (WGE‐SRH) Index (Moreau et al. 2020), the contraceptive existence of choice and the contraceptive exercise of choice subscales, in our survey to facilitate examination of concurrent construct validity with the Agency in Contraceptive Decisions Scale. We selected this measure a priori as a related measure of contraceptive empowerment. We included the index items that were validated in Nigeria and Uganda; due to an inadvertent omission of one item during survey development, this comprised eight of the nine items from the original contraceptive existence of choice and the contraceptive exercise of choice subscales.

#### Psychometric Analysis and Item Reduction

*Exploratory Factor Analysis*. We first examined item properties by looking at response frequencies by item as well as the mean and median scores on each item. To gain an impression of how the overall item pool performed, we also examined the internal consistency of the full item pool, including interitem correlations, item‐rest correlations, and excluded item alphas. To reduce the item pool and assess the dimensionality and structure of the Agency in Contraceptive Decisions Scale, we conducted an exploratory factor analysis (EFA). We first subset a random half of our sample (half of the sample from Nigeria and half of the sample from Uganda, *n* = 1501), retaining the second half for confirmatory factor analysis (CFA). We assessed the appropriateness of our data for factor analysis using the Kaiser—Meyer–Olkin (KMO) test for sampling adequacy, that is, a measure of the common variance among items (Kaiser 1974). This test operates under the assumption that having a lower proportion of common variance renders the data better suited to EFA (Kaiser 1974). We looked for a KMO value of >0.6.

Next, we used EFA to reach a set of items that maximized statistical efficiency and optimized coverage of constructs in our measurement framework (Figure 2). The psychometric analyses were conducted by an American researcher, and key decisions about item inclusion/exclusion and scale construction were only considered final after input from all team members was solicited. We conducted the process iteratively, repeating the following steps:

1. First, we used Horn's parallel analysis to produce adjusted eigenvalues, examining these along with a scree plot to determine the number of factors to retain (Horn 1965). In the scree test, we looked for the “elbow” of the plot to suggest how many factors to extract (Cattell 1966).
2. Next, using promax rotation and specifying the number of factors suggested by Horn's parallel analysis, we examined which items loaded onto which factors. For example, if in step one Horn's parallel analysis and scree plot suggested six factors, we would examine how items loaded onto six factors.
3. We removed items that did not load on any of the extracted factors at >0.5, as this indicates a weak relationship between the item and the underlying factors extracted. We also removed those that loaded on multiple factors, as this makes it difficult to interpret the factors (DeVellis 2017). In addition to considering these elements of statistical efficiency, we aimed to maintain domain and construct coverage when making decisions about what items to remove and retain. Thus, we retained specific items to ensure fidelity to and coverage of constructs in our measurement framework (Figure 2). For example, if an item did not load highly but its removal would mean a particular construct in the framework was not represented, we endeavored to retain that item.
4. After removing items in step 3, we reran the analyses with the refined set of items, starting again at step 1. We repeated these steps until we had a solution in which all items loaded onto a factor at >0.5, except in cases where an item was retained for construct coverage. During this iterative process, we also examined internal consistency reliability of the overall scale and potential subscales using Cronbach's α where needed to inform decisions about which items to drop/retain. We aimed to balance construct coverage and parsimony, so in some cases, we removed items loading >0.5 on a factor if there was ample coverage (at least two items) of that construct. These decisions were made based on which items had better phrasing and psychometric properties, with consensus among all members of the team.

*Confirmatory Factor Analysis*. To verify the factor structure proposed by the EFA, we used the second half of the sample (*n* = 1501) to perform CFA. We fit a structural equation model using maximum likelihood estimation and examined the fit indices, aiming for target values of Comparative FIt Index (CFI) ≥  0.95, Standardized Root Meant Square REsidual (SRMR) ≤  0.08, and Root Mean Square Error of Approximation (RMSEA) ≤  0.06 (DeVellis 2017). We also assessed the modification indices to assess whether additional parameters (path coefficients or covariances between items/constructs) needed to be estimated. We then respecified the model and examined fit indices of the final model to determine the extent to which the factor structure was compatible with the solution generated via EFA.

*Score Construction*. Responses for all items were coded as follows: “strongly no” = 0, “no” = 1, “yes” = 2, and “strongly yes” = 3, with a stronger affirmative response representing greater agency. Several negatively worded items were reverse‐coded to match this coding scheme. After the final item set was identified, we constructed overall scale and subscale scores using complete case analysis (i.e., only observations with valid responses on all items in the scale were included in the final scores). We then examined the correlation between all subscales to assess their suitability for a composite scale score. We examined descriptive statistics (mean, standard deviation, skew, kurtosis) for overall and subscale scores, each calculated using a mean of complete responses for relevant items on the 4‐point response scale.

*Concurrent Construct Validity Analysis*. To examine the concurrent validity of the Agency in Contraceptive Decisions Scale, we assessed the degree to which overall and subscale scores were associated with the WGE‐SRH contraceptive existence of choice and contraceptive exercise of choice subscale scores. This was chosen a priori as a measure of a similar construct to be used for validation. We regressed mean WGE‐SRH Index scores on mean scores of the Agency in Contraceptive Decisions Scale, accounting for geographic clustering (local government area level in Nigeria or district level in Uganda).

### Ethical Considerations

At the time of recruitment for all data collection procedures (cognitive interviews and surveys), participants were explained the purpose of the study and the expectations in terms of time and participation. Data collection procedures were only conducted after obtaining verbal consent from all participants. For any minors, assent was obtained to avoid any potential disclosure of the subject's current/past sexual activity and/or use of or interest in contraception. All interviews and surveys were conducted in private, confidential spaces identified by the participants. All data were identified only with unique ID numbers and housed in secure password‐protected folders. Only study investigators and study personnel had access to the data files. All consent, data collection, and data management procedures including for cognitive interviews and surveys were approved by the UCSF (IRB #21‐34470) and Makerere School of Public Health (SPH‐2022‐212) Institutional Review Boards, the Uganda National Council for Science and Technology (HS1087ES), and the National Health Research Ethics Committee, Nigeria (NHREC/01/01/2007‐25/09/2020).

## RESULTS

### Item Generation and Refinement

Initial results from cognitive interviews inspired several high‐level changes. Notably, when cognitive interviewers asked them to affirm or deny first‐person statements (e.g., “I believe the government should provide methods to avoid pregnancy for free.”), participants often did not reflect on their own opinions or experiences. Instead, they answered questions generally or hypothetically. After all items were rephrased as from first‐person “I” statements to second‐person questions (e.g., “Do you believe the government should provide methods to avoid pregnancy for free?”), participants’ responses better reflected their own opinions and experiences. Additionally, interview results demonstrated possible misinterpretation of the phrase “pregnancy prevention,” which we chose to use over “family planning” or “contraception” to include contraceptive use outside the context of planning one's family and traditional methods of pregnancy prevention. “Pregnancy prevention” was interpreted by some to include abortion; thus, we rephrased to “avoiding pregnancy” and clarified our focus on prevention in the introduction. Cognitive interviews also helped refine the phrasing of individual items to ensure that items were phrased to be uniformly interpreted. Finally, we modified Likert scale response options based on results from the cognitive interviews to ensure participants used the full range of response options. Offering response options in a two‐step process was an important innovation that came about as a result of our cognitive interviews. While we originally had a more traditional Likert scale response set, during interviews, RAs noticed participants would frequently respond with a simple “yes” or “no” and then specify the strength of their response with probing. The final response set included: (1) strongly no, (2) no, (3) yes, (4) strongly yes, and was administered by first asking participants to say yes or no, then prompting whether it was a strong yes or no.

### Item Testing and Reduction

#### Survey Sample Characteristics

Overall, we had a sample of *N* = 3002 participants across study sites in Nigeria (*n* = 580) and Uganda (*n* = 2422). Participants’ mean age was 27.6 years (Table 1), with a slightly higher mean age in Nigeria than Uganda (31.7 vs. 26.6). Participants had a mean of 4.7 children, with participants in Uganda having a greater mean number than in Nigeria (5.0 vs. 3.4). Most women reported identifying as Christian, though in Uganda, 20.9 percent of the sample were Muslim. Education levels were higher in Nigeria compared to Uganda (80 percent of the Nigerian sample completed secondary school or more, compared to only 27 percent in Uganda). Across both countries, most women were married or partnered (96.2 percent).

**TABLE 1:** Survey participant characteristics, overall and by country

Characteristic	Overall	Nigeria	Uganda
	Mean (SD)	Mean (SD)	Mean (SD)
*N*s	3002	580	2422
Age	27.6 (6.8)	31.7 (6.8)	26.6 (6.5)
Parity	4.7 (1.6)	3.4 (1.6)	5.0 (1.4)

	*N* (%)	*N* (%)	*N* (%)
Current contraceptive use			
Yes	2481 (82.6)	580 (100.0)	1901 (78.5)
No	521 (17.4)	0 (0.0)	521 (21.5)
Religion			
Christianity	2484 (82.7)	574 (99.0)	1910 (78.9)
Islam	509 (17.0)	2 (0.3)	507 (20.9)
Other	9 (0.3)	4 (0.7)	5 (0.21)
Education			
None/less than primary	143 (4.8)	12 (2.1)	131 (5.4)
Primary	1749 (58.3)	107 (18.5)	1642 (67.8)
Secondary	883 (29.4)	333 (57.4)	550 (22.7)
College/university	227 (7.6)	128 (22.1)	99 (4.1)
Marital status			
Married/partnered	2888 (96.2)	527 (90.9)	2361 (97.5)
Not currently married/partnered	74 (2.5)	34 (5.9)	40 (1.7)
Never married	40 (1.3)	19 (3.3)	21 (0.9)

#### Exploratory Factor Analysis and Item Reduction

The iterative item reduction process resulted in a 15‐item scale with four subscales: (1) Beliefs about Rights and Perceived Decision‐making Control, (2) Decision‐making Self‐efficacy, (3) Knowledge Aligned with Preferences, and (4) Control over Use or Non‐use. Subscales 1–3 represent constructs from Domain 1 of the Contraceptive Agency Framework (agency in making contraceptive decisions), while Subscale 4 relates to Domain 2 (agency in acting on contraceptive decisions) (Figure 2). The Beliefs about Rights and Perceived Decision‐making Control subscale includes eight items that pertain to the support/information, consciousness of rights, critical reflection, and perceived control aspects of the Contraceptive Agency Framework. The Decision‐making Self‐efficacy subscale includes three items corresponding to the self‐efficacy construct in the framework. The Knowledge Aligned with Preferences subscale includes two items that represent the information‐in‐line‐with‐one's‐preferences aspect of the framework. Finally, the Control Over Use or Non‐use subscale includes two items representing the exercise of choice aspect of the framework. The 15 items and their dominant factor loading for the final scale are displayed in Table 2. All but one item, retained for content validity as it is the only item that represents the critical reflection construct, loaded greater than 0.5 on its dominant factor.

**TABLE 2:** Final Agency in Contraceptive Decisions Scale with results from exploratory and confirmatory factor analyses in Nigeria and Uganda (*N* = 3002)

			EFA (*N* = 1501)	CFA (*N* = 1501)		
#	Item	Mean (SD)	Rotated factor loading	Standardized coefficient	*p* > *z*	95% CI
Overall Scale: mean = 2.6, min = 0.9, max = 3.0, SD = 0.4, α = 0.8						
Subscale 1: Beliefs about Rights and Perceived Decision‐making Control (related to what, if anything, to do to avoid pregnancy); mean = 2.7, min = 1.3, max = 3.0, SD = 0.4, α = 0.8						
1	Could you get a method to avoid pregnancy if you needed it?	2.7 (0.56)	0.6	0.8	<0.001	(0.7, 0.8)
2	Could you talk to a healthcare provider about ways to avoid pregnancy if you wanted to?	2.8 (0.5)	0.7	0.7	<0.001	(0.7, 0.8)
3	Do you believe the government should provide methods to avoid pregnancy for free?	2.9 (0.3)	0.6	0.7	<0.001	(0.6, 0.7)
4	Should people have the right to be told about the available ways to avoid pregnancy?	2.8 (0.4)	0.8	0.6	<0.001	(0.6, 0.7)
5	Should people have the right to choose from different ways to avoid pregnancy if they want to do so?	2.8 (0.4)	0.8	0.7	<0.001	(0.6, 0.7)
6	Do you believe that women's choices about avoiding pregnancy are unfairly limited because of community views of women?	2.3 (1.0)	0.2	0.2	<0.001	(0.1, 0.2)
7	Is it your decision who you tell or do not tell about if you are doing something to avoid pregnancy?	2.7 (0.5)	0.6	0.7	<0.001	(0.7, 0.8)
8	Do you decide for yourself whose opinions you listen to about avoiding pregnancy?	2.7 (0.5)	0.5	0.7	<0.001	(0.7, 0.8)
Subscale 2: Decision‐making Self‐efficacy (related to what, if anything, to do to avoid pregnancy); mean = 2.6, min = 0.0, max = 3.0, SD = 0.6, α = 0.8						
9	If a family member did not support your choice, would you still be able to do what is best for you, related to avoiding pregnancy?	2.6 (0.8)	0.8	0.7	<0.001	(0.7, 0.7)
10	Are you confident you can do what is best for you related to avoiding pregnancy?	2.7 (0.6)	0.7	0.9	<0.001	(0.8, 0.9)
11	If something or someone stood in your way, could you find a way to do what you want related to avoiding pregnancy?	2.6 (0.7)	0.6	0.8	<0.001	(0.7, 0.8)
Subscale 3: Knowledge Aligned with Preferences (related to what, if anything, to do to avoid pregnancy); mean = 2.4, min = 0.0, max = 3.0, SD = 0.7, α = 0.8						
12	Do you know different ways to avoid pregnancy?	2.4 (0.8)	0.7	0.8	<0.001	(0.8, 0.8)
13	At this point in your life, do you know what you want to know about avoiding pregnancy?	2.4 (0.8)	0.7	0.8	<0.001	(0.8, 0.9)
Subscale 4: Control over Use or Non‐use (interference with one's ability to act on decisions related to what, if anything, to do to avoid pregnancy); mean = 2.5, min = 0.0, max = 3.0, SD = 0.8, α = 0.8						
14	Is someone in your life making you do something you don't want to do related to avoiding pregnancy?	2.5 (0.9)	0.7	0.9	<0.001	(0.8, 0.9)
15	Is someone in your life stopping you from doing something you want related to avoiding pregnancy?	2.5 (0.9)	0.7	0.8	<0.001	(0.7, 0.9)
Subscale covariances (from CFA)						
Covariance (Beliefs about Rights and Perceived Decision‐making Control, Decision‐making Self‐efficacy)				0.7	<0.001	(0.7, 0.7)
Covariance (Beliefs about Rights and Perceived Decision‐making Control, Knowledge Aligned with Preferences)				0.5	<0.001	(0.4, 0.5)
Covariance (Beliefs about Rights and Perceived Decision‐making Control, Control Over Use or Non‐use)				0.2	<0.001	(0.2, 0.3)
Covariance (Decision‐making Self‐efficacy, Knowledge Aligned with Preferences)				0.3	<0.001	(0.3, 0.4)
Covariance (Decision‐making Self‐efficacy, Control Over Use or Non‐use)				0.2	<0.001	(0.2, 0.3)
Covariance (Knowledge Aligned with Preferences, Control Over Use or Non‐use)				0.04	0.19	(−0.02, 1.1)

#### Item Response Means

Table 2 shows the mean responses on the final set of items retained for the Agency in the Contraceptive Decisions Scale. Means of the remainder of the items not included in the final scale can be found in Online Appendix 2. Item scores ranged from 2.3 to 2.9, with a higher score representing higher agency.

#### Reliability

Cronbach's α for the 15‐item Agency in Contraceptive Decisions Scale and all subscales was 0.8 or greater (Table 2).

*Confirmatory Factor Analysis*. The structural equation model we used for CFA included the 15 items corresponding to the four factors extracted by the EFA. The initial CFA model showed moderate support for the four‐factor solution (CFI: 0.9, SRMR: 0.05, RMSEA: 0.08). Modification indices showed that accounting for additional covariances between items, as specified in the bottom panel of Table 2, would improve the model fit. After respecifying the model to include those additional covariances, fit indices improved to support the four‐factor solution (CFI: 1.0, SRMR: 0.04, RMSEA: 0.06). Items showed strong significance (*p* < 0.001) and high factor loadings (all standardized coefficients >0.6 except item #6, which was retained for construct coverage).

#### Final Scale and Subscale Scores

*N* = 2963 observations were included in the final overall scale and subscale score construction. *N* = 39 (1.3 percent) were excluded due to missingness on any of the 15 final items of the scale. In the sample with complete data on the final items included in the Agency in Contraceptive Decisions Scale (*n* = 2963), scores were calculated for those with complete responses as the mean of all 15 items and ranged from 0.9 to 3.0 with an overall mean of 2.6. Overall scores and subscale scores are presented by country, contraceptive use status, and additional demographic characteristics in Table 3. Mean scores were similar across Nigeria and Uganda (2.7 vs. 2.6, respectively) and across contraceptive users and nonusers (2.6 vs. 2.5, respectively). Scores on the Beliefs about Rights and Perceived Decision‐making Control subscale ranged from 1.3 to 3.0, with an overall mean of 2.7 while the scores on the Decision‐making Self‐efficacy, Contraceptive Knowledge, and Control Over Use or Non‐use subscales ranged from 0 to 3, with means of 2.6, 2.4, and 2.5, respectively.

**TABLE 3:** Agency in Contraceptive Decisions Scale scores by country and participant characteristics (*N* = 2963)^a^

	Overall	Subscale 1	Subscale 2	Subscale 3	Subscale 4
	Mean (SD)	Mean (SD)	Mean (SD)	Mean (SD)	Mean (SD)
Country					
Nigeria	2.7 (0.3)	2.7 (0.3)	2.8 (0.4)	2.4 (0.8)	2.8 (0.4)
Uganda	2.6 (0.4)	2.7 (0.4)	2.6 (0.6)	2.4 (0.7)	2.4 (0.9)
Contraceptive use					
Yes	2.6 (0.3)	2.7 (0.4)	2.7 (0.5)	2.4 (0.7)	2.5 (0.8)
No (Uganda only)	2.5 (0.4)	2.7 (0.4)	2.3 (0.7)	2.2 (0.8)	2.5 (0.8)
Age					
≤25	2.6 (0.4)	2.7 (0.4)	2.5 (0.6)	2.3 (0.8)	2.5 (0.8)
26+	2.7 (0.3)	2.7 (0.3)	2.7 (0.5)	2.5 (0.7)	2.5 (0.8)
Parity					
≤4	2.6 (0.3)	2.7 (0.4)	2.6 (0.5)	2.4 (0.8)	2.5 (0.7)
5+	2.6 (0.4)	2.7 (0.4)	2.6 (0.6)	2.4 (0.7)	2.4 (0.9)
Religion					
Christianity	2.6 (0.4)	2.7 (0.4)	2.6 (0.6)	2.3 (0.8)	2.5 (0.7)
Islam	2.7 (0.3)	2.8 (0.3)	2.8 (0.5)	2.6 (0.5)	2.3 (1.1)
Other	2.7 (0.2)	2.8 (0.3)	2.5 (0.7)	2.7 (0.4)	2.6 (0.7)
Education					
None/less than primary	2.7 (0.3)	2.8 (0.3)	2.7 (0.5)	2.4 (0.8)	2.4 (0.8)
Primary	2.6 (0.4)	2.7 (0.4)	2.5 (0.6)	2.3 (0.8)	2.4 (0.8)
Secondary	2.7 (0.3)	2.8 (0.3)	2.7 (0.5)	2.6 (0.6)	2.6 (0.8)
College/university	2.8 (0.2)	2.8 (0.2)	2.8 (0.3)	2.6 (0.6)	2.7 (0.6)
Marital status					
Married/partnered	2.6 (0.4)	2.7 (0.4)	2.6 (0.6)	2.4 (0.7)	2.5 (0.8)
Not currently married/partnered	2.6 (0.3)	2.7 (0.3)	2.6 (0.5)	2.3 (0.7)	2.6 (0.8)
Never married	2.8 (0.2)	2.8 (0.2)	2.9 (0.2)	2.5 (0.7)	2.7 (0.7)

^a^From this stage onwards, the *N*s represent complete cases (i.e., those that had valid responses to all 15 items in the final scale.)

Correlations between subscales 1–3 were moderate. The Beliefs about Rights and Perceived Decision‐making Control subscale was moderately correlated with the Decision‐making Self‐efficacy and Knowledge Aligned with Preferences subscales (Pearson coefficient = 0.5 and 0.4, respectively). The Decision‐making Self‐efficacy subscale correlated 0.3 with the Knowledge Aligned with Preferences subscale. Correlations of subscales 1–3 with subscale 4 were low: the Beliefs about Rights and Perceived Decision‐making Control, Decision‐making Self‐efficacy, and Knowledge Aligned with Preferences subscales had Pearson coefficients all < 0.2 with the Control Over Use or Non‐use subscale, which was the only subscale containing items from Domain 2 of the measurement framework (related to *acting* on decisions).

### Concurrent Validity Analysis

Overall Agency in Contraceptive Decisions Scale scores were found to be significantly positively associated with the WGE‐SRH contraceptive existence of choice and contraceptive exercise of choice subscales (β = 1.2, 95 percent CI: 0.9, 1.5) (Table 4). Additionally, all subscale scores were significantly positively associated with the WGE‐SRH contraceptive existence of choice and contraceptive exercise of choice subscales (Beliefs about Rights and Perceived Decision‐making Control—β = 0.9, 95 percent CI: 0.7, 1.0; Decision‐making Self‐efficacy—β = 0.5, 95 percent CI: 0.3, 0.7; Knowledge Aligned with Preferences—β = 0.3, 95 percent CI: 0.2, 0.5; Control Over Use or Non‐use—β = 0.4, 95 percent CI: 0.2, 0.6).

**TABLE 4:** Association of Agency in Contraceptive Decisions Scale and subscale scores with WGE‐SRH Index: Results from unadjusted linear regression models (*N* = 2963)

		WGE‐SRH Score
		Coefficient (95% CI) *p*‐value
Overall score	Agency in Contraceptive Decisions Scale	1.2 (0.9, 1.5) *p* < 0.001
Subscale scores	1. Beliefs about Rights and Perceived Decision‐making Control	0.9 (0.7, 1.0) *p* < 0.001
	2. Decision‐making Self‐efficacy	0.5 (0.3, 0.7) *p* = 0.001
	3. Knowledge Aligned with Preferences	0.3 (0.2, 0.5) *p* < 0.001
	4. Control Over Use or Non‐use	0.4 (0.2, 0.6) *p* = 0.005

NOTES: Due to one item being inadvertently omitted from the survey instrument, the WGE‐SRH Index score was constructed using eight of nine items included in the cross‐site measure covering the existence and exercise of contraceptive choice. Results are from linear regression models accounting for geographic clustering at the Local government area level in Nigeria and the district level in Uganda; Agency in Contraceptive Decisions Scale scores as predictors and WGE‐SRH Index Scores as outcome.

Models include all observations with complete scores on the final Agency in Contraceptive Decisions Scale and all subscales.

## DISCUSSION

We developed and validated the Agency in Contraceptive Decisions Scale in Nigeria and Uganda based on the theoretically informed Contraceptive Agency Framework (Holt et al. 2024). The scale includes 15 items representing four subscales: Beliefs about Rights and Perceived Decision‐making Control, Decision‐making Self‐efficacy, Knowledge Aligned with Preferences, and Control Over Use or Non‐use. The Agency in Contraceptive Decisions Scale provides an overall measure of an individual's agency in decision‐making and actions related to pregnancy prevention.

This new rights‐based measure fills an important gap in the contraception measurement ecosystem. Importantly, the Agency in Contraceptive Decisions Scale does not privilege any particular decision or behavior regarding contraception as the right one. In contrast to other measures of contraceptive decision‐making, the Agency in Contraceptive Decisions Scale includes items reflecting the degree to which individuals have agency in deciding *not* to use contraception (Figure 2) through item wording that does not equate contraceptive nonuse or covert use with disempowerment, such as the item “Is someone in your life making you do something you don't want to do related to avoiding pregnancy?” Additionally, the Knowledge Aligned with Preferences subscale captures whether people have the information they desire to make choices about pregnancy prevention, not specifically to enact decisions related to contraceptive use. Further, it does not impose any external threshold for the level of information required to make an informed choice, but rather allows this to be self‐defined. The scale also uniquely includes an item on critical reflection—an often‐overlooked construct in existing measures—and includes items such as “Do you decide for yourself whose opinions you listen to about avoiding pregnancy?” that interrogate potential sources of social influence beyond partners. Another strength of the measure is that it was developed to be gender neutral, and cognitive interviews with men influenced item refinement. While we only tested the scale with women in the present study, we recommend future testing among a broader population.

Given the length of the Agency in Contraceptive Decisions Scale (15 items), we anticipate it will be most useful for application in program evaluations and other special studies. Research using the Agency in Contraceptive Decisions Scale can guide social and behavior change programs to focus on improving salient agency‐related constructs among clients rather than focusing on promoting contraceptive uptake. As such, it can further a rights‐based approach to contraceptive programming that acknowledges and respects the range of empowered decisions a person may make related to contraception. The four subscales of the Agency in Contraceptive Decisions Scale can be utilized alone or as part of a composite, depending on outcomes of interest and researchers’ priorities. Since the Control Over Use or Non‐use subscale was not highly correlated with the other subscales, researchers can consider creating a composite score without this subscale if they see fit. However, the high alpha of the 15‐item scale and its association with the previously validated WGE‐SRH Index suggest it is reasonable to treat the full scale as a composite measure of contraceptive agency.

It is also worth noting that our item generation and refinement processes involved rigorous foundational work that is essential to developing valid measures of complex constructs, but is often undervalued (Boateng et al. 2018; DeVellis 2017). Our cross‐country efforts to hone the item pool were a major strength of the process and allowed comprehensive reflections on the applicability and phrasing of all items. Subsequently, our collaborative development of the cognitive interview training resulted in tailored materials that ensured high‐quality interviews and increased familiarity with this niche method among local researchers. This is all the more important given that recent work has demonstrated a range of question failures plaguing global public health research, from simple concerns of question sequencing to more complex concerns around resonance (Scott et al. 2019). Without engaging interdisciplinary, cross‐cultural, and language‐diverse perspectives early in item pool development and without a rigorous cognitive interview process, scale items would have succumbed to major misinterpretation. Future measure development efforts and research dissemination may benefit from enhanced attention to and transparency around these important formative steps.

We acknowledge several limitations in the development of the Agency in Contraceptive Decisions Scale. First, the survey sample in Nigeria was restricted to contraceptive users. The Nigerian sample was diverse in terms of the range of methods used and participant ages, and our overall sample is bolstered by a large and more diverse sample of women in Uganda, inclusive of contraceptive users and nonusers from a range of different backgrounds. However, future work is warranted to confirm that the scale is valid for use in measuring agency among nonusers of contraception in Nigeria. Our measurement framework (Holt et al. 2024) included two constructs that proved especially difficult to measure: (1) clarity of one's values—to be clear about one's personal values related to preventing pregnancy and (2) critical reflection—awareness of intersecting social constructions that can constrain or enhance individuals’ ability to exercise the right to pregnancy prevention choices. While ultimately all items related to clarity of values fell out during psychometric analysis, we chose to retain one item related to critical reflection that fell below our a priori factor loading threshold, as this is a particularly important innovation of the measure. It is possible that the value of different constructs shifts depending on the cultural context. Relatedly, this measure was developed and tested in Nigeria and Uganda, so it may not be generalizable to other contexts exactly as developed. While we anticipate that the items included in the measure are generally relevant, exact interpretations may vary depending on language, cultural context, etc., and researchers should assess validity for use in other contexts where feasible. Additionally, due to time and resource constraints, we were unable to conduct test–retest reliability assessments to ensure participant responses were stable over time. It should be noted that agency is a complex, multifaceted, and dynamic construct, so it is difficult to assert that responses would have been stable upon retest. Finally, our measure of correlational validity, the combined WGE‐SRH contraceptive existence of choice and contraceptive exercise of choice subscale scores (Moreau et al. 2020), was constructed using only eight of the nine items from the initial measure, due to an inadvertent omission in the survey development process. However, the significant associations found with the overall Agency in Contraceptive Decisions Scale scores and each subscale score provide compelling evidence that this is a valid measure of agency in making and acting on contraceptive decisions. Future longitudinal research can examine the predictive validity of this scale to further assess its utility as a key measure of success in contraceptive programming.

## CONCLUSION

We found evidence that the Agency in Contraceptive Decisions Scale is valid and reliable for use in measuring the latent construct of contraceptive agency among women of reproductive age in Nigeria and Uganda. The composite scale and individual subscales can be used to capture individuals’ ability to make and act on contraceptive decisions, regardless of what those decisions are, and expand our understanding of individuals’ contraceptive decision‐making empowerment. This widely applicable rights‐based measure offers researchers and program evaluators a valuable tool with which to assess the degree to which programs, policies, and service provision support all people to make empowered contraceptive decisions.

## CONFLICT OF INTEREST STATEMENT

The authors declare that they have no competing interests.

## ETHICS STATEMENT

This study and all related procedures were approved by the Institutional Review Board of the University of California, San Francisco (IRB #21‐34470), and Makerere University School of Public Health Institutional Review Boards (SPH‐2022‐212); the Uganda National Council for Science and Technology (HS1087ES); and the National Health Research Ethics Committee, Nigeria (NHREC/01/01/2007‐25/09/2020)

## PATIENT CONSENT STATEMENT

Informed consent to publish anonymized data from the research was obtained from all participants recruited to the study.

## PERMISSION TO REPRODUCE MATERIAL

We have obtained permission to reproduce Figure 2 from Dr. Kelsey Holt, the first author of “Conceptualizing Contraceptive Agency: A Critical Step to Enable Human Rights‐Based Family Planning Programs and Measurement,” published in *Global Health: Science and Practice* (https://doi.org/10.9745/GHSP‐D‐23‐002990).

## Supporting information



Appedix 1

Appedix 2

## Data Availability

The dataset used and/or analyzed during the current study is available from the corresponding author on reasonable request.
