# Supplementary material for: Development and Validation of the Agency in Contraceptive Decisions Scale in Uganda and Nigeria
Source: Stud Fam Plann. 2025 Sep 22;56(3):655–75. doi: 10.1111/sifp.70033 (PMC12501711; doi:10.1111/sifp.70033)
Supplement: Supplementary file 1 — Appedix 1 [file SIFP-56-655-s001.docx]

**ITEM POOL**

Now we are going to move on to the part where we will test the wording on a list of questions to help us understand how we can improve the questions so they can be understood by many people. All of the questions I will be reading, will ask about you and your personal situation. We want to know about your own choices and needs in your own life. As a reminder, these questions will help us to understand people’s choices around contraception. All of the questions I will read are about avoiding pregnancy, making sure it does not happen at all – not about removing or washing away the pregnancy after it has happened. Do you understand me?

Read aloud, “Yes or no” followed by asking “No or strong no” OR “Yes or strong yes”.

**Questionnaire I: #1-32 (ODK will randomly select respondent for Questionnaire I or Questionnaire II)**

| **#** | **Item** | **Response Option** | **Questions/Probes** |
| --- | --- | --- | --- |
| **Domain 1.** | | | |
|  | Each of the following statements may or may not apply to you. Please rate how much you agree or disagree with the following statements.  If you are unsure about how to answer any items, please give your best guess. | | |
|  | Do you know what you want to do regarding avoiding or not avoiding pregnancy, that is, making sure it does not happen when you do not want it to? | Strongly no 1  No 2  Yes 3  Strongly yes 4  I don’t understand 5 | 1. **Please tell me why you selected that answer.** 2. **Was the question confusing or difficult to answer in any way?**    1. [IF YES]: What specifically made the question confusing or difficult to answer? 3. ***IF THEY DON’T UNDERSTAND, REPEAT THE QUESTION AND***What specifically made the question confusing or difficult to answer? |
|  | Do you know what your options are for avoiding pregnancy, that is, making sure it does not happen when you do not want it to? | Strongly no 1  No 2  Yes 3  Strongly yes 4  I don’t understand 5 | 1. **Please tell me why you selected that answer.** 2. **Was the question confusing or difficult to answer in any way?**    1. [IF YES]: What specifically made the question confusing or difficult to answer? 3. ***IF THEY DON’T UNDERSTAND, REPEAT THE QUESTION AND***What specifically made the question confusing or difficult to answer? |
|  | Do you know what you need related to avoiding or not avoiding pregnancy? | Strongly no 1  No 2  Yes 3  Strongly yes 4  I don’t understand 5 | 1. **Please tell me why you selected that answer.** 2. **Was the question confusing or difficult to answer in any way?**    1. [IF YES]: What specifically made the question confusing or difficult to answer? 3. ***IF THEY DON’T UNDERSTAND, REPEAT THE QUESTION AND***What specifically made the question confusing or difficult to answer? |
|  | Do you know if you want to be doing something to avoid pregnancy? | Strongly no 1  No 2  Yes 3  Strongly yes 4  I don’t understand 5 | 1. **Please tell me why you selected that answer.** 2. **Was the question confusing or difficult to answer in any way?**    1. [IF YES]: What specifically made the question confusing or difficult to answer? 3. ***IF THEY DON’T UNDERSTAND, REPEAT THE QUESTION AND***What specifically made the question confusing or difficult to answer? |
|  | If you wanted to use a method to avoid getting pregnant, do you know how to get it? | Strongly no 1  No 2  Yes 3  Strongly yes 4  I don’t understand 5 | 1. **Please tell me why you selected that answer.** 2. **Was the question confusing or difficult to answer in any way?**    1. [IF YES]: What specifically made the question confusing or difficult to answer? 3. ***IF THEY DON’T UNDERSTAND, REPEAT THE QUESTION AND***What specifically made the question confusing or difficult to answer? |
|  | Do you know everything you want to know about your options to avoid pregnancy? | Strongly no 1  No 2  Yes 3  Strongly yes 4  I don’t understand 5 | 1. **Please tell me why you selected that answer.** 2. **Was the question confusing or difficult to answer in any way?**    1. [IF YES]: What specifically made the question confusing or difficult to answer? 3. ***IF THEY DON’T UNDERSTAND, REPEAT THE QUESTION AND***What specifically made the question confusing or difficult to answer? |
|  | Do you feel you have enough support from people in your life to be able to make decisions about avoiding pregnancy? | Strongly no 1  No 2  Yes 3  Strongly yes 4  I don’t understand 5 | 1. **Please tell me why you selected that answer.** 2. **Was the question confusing or difficult to answer in any way?**    1. [IF YES]: What specifically made the question confusing or difficult to answer? 3. ***IF THEY DON’T UNDERSTAND, REPEAT THE QUESTION AND***What specifically made the question confusing or difficult to answer? |
|  | Do you feel clear about who you want involved in your decisions about doing or not doing something to avoid pregnancy? | Strongly no 1  No 2  Yes 3  Strongly yes 4  I don’t understand 5 | 1. **Please tell me why you selected that answer.** 2. **Was the question confusing or difficult to answer in any way?**    1. [IF YES]: What specifically made the question confusing or difficult to answer? 3. ***IF THEY DON’T UNDERSTAND, REPEAT THE QUESTION AND***What specifically made the question confusing or difficult to answer? |
|  | Do you believe it is your right to use or not use a method to avoid pregnancy according to your wishes? | Strongly no 1  No 2  Yes 3  Strongly yes 4  I don’t understand 5 | 1. **Please tell me why you selected that answer.** 2. **Was the question confusing or difficult to answer in any way?**    1. [IF YES]: What specifically made the question confusing or difficult to answer? 3. ***IF THEY DON’T UNDERSTAND, REPEAT THE QUESTION AND***What specifically made the question confusing or difficult to answer? |
|  | Do you believe everyone should be able to use a method to avoid getting pregnant when they want to? | Strongly no 1  No 2  Yes 3  Strongly yes 4  I don’t understand 5 | 1. **Please tell me why you selected that answer.** 2. **Was the question confusing or difficult to answer in any way?**    1. [IF YES]: What specifically made the question confusing or difficult to answer? 3. ***IF THEY DON’T UNDERSTAND, REPEAT THE QUESTION AND***What specifically made the question confusing or difficult to answer? |
|  | Do you think everyone should be able to choose to stop using a method to avoid pregnancy if they don’t want to use one? | Strongly no 1  No 2  Yes 3  Strongly yes 4  I don’t understand 5 | 1. **Please tell me why you selected that answer?**    1. Can you please use your own words to tell me what you think this question was asking? 2. **Was the question confusing or difficult to answer in any way?**    1. [IF YES]: What specifically made the question confusing or difficult to answer? 3. ***IF THEY DON’T UNDERSTAND, REPEAT THE QUESTION AND***What specifically made the question confusing or difficult to answer? |
|  | Do you feel you have the right to refuse a healthcare worker who tries to make you use a method to avoid pregnancy? | Strongly no 1  No 2  Yes 3  Strongly yes 4  I don’t understand 5 | 1. **Please tell me why you selected that answer.** 2. **Was the question confusing or difficult to answer in any way?**    1. [IF YES]: What specifically made the question confusing or difficult to answer? 3. ***IF THEY DON’T UNDERSTAND, REPEAT THE QUESTION AND***What specifically made the question confusing or difficult to answer? |
|  | Do you believe you have the right to use a method to avoid pregnancy even if a partner or family member tries to keep you from doing so? | Strongly no 1  No 2  Yes 3  Strongly yes 4  I don’t understand 5 | 1. **Please tell me why you selected that answer.** 2. **Was the question confusing or difficult to answer in any way?**    1. [IF YES]: What specifically made the question confusing or difficult to answer? 3. ***IF THEY DON’T UNDERSTAND, REPEAT THE QUESTION AND***What specifically made the question confusing or difficult to answer? |
|  | Should the choice about using or not using a method to avoid pregnancy be your own to make even if other people disagree? | Strongly no 1  No 2  Yes 3  Strongly yes 4  I don’t understand 5 | 1. **Please tell me why you selected that answer.** 2. **Was the question confusing or difficult to answer in any way?**    1. [IF YES]: What specifically made the question confusing or difficult to answer? 3. ***IF THEY DON’T UNDERSTAND, REPEAT THE QUESTION AND***What specifically made the question confusing or difficult to answer? |
|  | Should people be able to use methods to avoid getting pregnant without their partners knowing if that’s what they want? | Strongly no 1  No 2  Yes 3  Strongly yes 4  I don’t understand 5 | 1. **Please tell me why you selected that answer.** 2. **Was the question confusing or difficult to answer in any way?**    1. [IF YES]: What specifically made the question confusing or difficult to answer? 3. ***IF THEY DON’T UNDERSTAND, REPEAT THE QUESTION AND***What specifically made the question confusing or difficult to answer? |
|  | Do you believe you have the right to access methods to avoid pregnancy at low prices? | Strongly no 1  No 2  Yes 3  Strongly yes 4  I don’t understand 5 | 1. **Please tell me why you selected that answer.** 2. **Was the question confusing or difficult to answer in any way?**    1. [IF YES]: What specifically made the question confusing or difficult to answer? 3. ***IF THEY DON’T UNDERSTAND, REPEAT THE QUESTION AND***What specifically made the question confusing or difficult to answer? |
|  | Do yo think the government should ensure availability of methods to avoid pregnancy at low prices? | Strongly no 1  No 2  Yes 3  Strongly yes 4  I don’t understand 5 | 1. **Please tell me why you selected that answer.** 2. **Was the question confusing or difficult to answer in any way?**    1. [IF YES]: What specifically made the question confusing or difficult to answer? 3. ***IF THEY DON’T UNDERSTAND, REPEAT THE QUESTION AND***What specifically made the question confusing or difficult to answer? |
| **Domain 2** | | | |
|  | **Instructions:** Read aloud all four response options “No, not at all; Yes, a little; Yes, somewhat; Yes, very well/very much”. | |  |
|  | Do you know how to overcome challenges that stand between you and what you want related to avoiding pregnancy? | No, not at all 1  Yes, a little 2  Yes, somewhat (as in more than a little) 3  Yes, very well/very much 4  I don’t understand 5 | 1. **Please tell me why you selected that answer.** 2. **Was the question confusing or difficult to answer in any way?**    1. [IF YES]: What specifically made the question confusing or difficult to answer? 3. ***IF THEY DON’T UNDERSTAND, REPEAT THE QUESTION AND***What specifically made the question confusing or difficult to answer? |
|  | Would you be able to resist if someone tried to make you use a method to avoid pregnancy when you did not want to? | No, not at all 1  Yes, a little 2  Yes, somewhat (as in more than a little) 3  Yes, very well/very much 4  I don’t understand 5 | 1. **Please tell me why you selected that answer.** 2. **Was the question confusing or difficult to answer in any way?**    1. [IF YES]: What specifically made the question confusing or difficult to answer? 3. ***IF THEY DON’T UNDERSTAND, REPEAT THE QUESTION AND***What specifically made the question confusing or difficult to answer? |
|  | Could you use a method to avoid pregnancy even if a partner or other family member doesn’t want you to? | No, not at all 1  Yes, a little 2  Yes, somewhat (as in more than a little) 3  Yes, very well/very much 4  I don’t understand 5 | 1. **Please tell me why you selected that answer.** 2. **Was the question confusing or difficult to answer in any way?**    1. [IF YES]: What specifically made the question confusing or difficult to answer? 3. ***IF THEY DON’T UNDERSTAND, REPEAT THE QUESTION AND***What specifically made the question confusing or difficult to answer? |
|  | Could you get a method to avoid pregnancy without anyone knowing if you wanted to? | No, not at all 1  Yes, a little 2  Yes, somewhat (as in more than a little) 3  Yes, very well/very much 4  I don’t understand 5 | 1. **Please tell me why you selected that answer.** 2. **Was the question confusing or difficult to answer in any way?**    1. [IF YES]: What specifically made the question confusing or difficult to answer? 3. ***IF THEY DON’T UNDERSTAND, REPEAT THE QUESTION AND***What specifically made the question confusing or difficult to answer? |
|  | In your opinion, is it ok for someone like you to use a method to avoid pregnancy if they want to? | No, not at all 1  Yes, a little 2  Yes, somewhat (as in more than a little) 3  Yes, very well/very much 4  I don’t understand 5 | 1. **Please tell me why you selected that answer.** 2. **Was the question confusing or difficult to answer in any way?**    1. [IF YES]: What specifically made the question confusing or difficult to answer? 3. ***IF THEY DON’T UNDERSTAND, REPEAT THE QUESTION AND***What specifically made the question confusing or difficult to answer? |
|  | In your opinion, is it ok for someone like you **NOT** to use a method to avoid pregnancy if they do not want to? | No, not at all 1  Yes, a little 2  Yes, somewhat (as in more than a little) 3  Yes, very well/very much 4  I don’t understand 5 | 1. **Please tell me why you selected that answer.** 2. **Was the question confusing or difficult to answer in any way?**    1. [IF YES]: What specifically made the question confusing or difficult to answer? 3. ***IF THEY DON’T UNDERSTAND, REPEAT THE QUESTION AND***What specifically made the question confusing or difficult to answer? |
|  | Do you feel confident about what you are doing or not doing to avoid pregnancy? | No, not at all 1  Yes, a little 2  Yes, somewhat (as in more than a little) 3  Yes, very well/very much 4  I don’t understand 5 | 1. **Please tell me why you selected that answer.** 2. **Was the question confusing or difficult to answer in any way?**    1. [IF YES]: What specifically made the question confusing or difficult to answer? 3. ***IF THEY DON’T UNDERSTAND, REPEAT THE QUESTION AND***What specifically made the question confusing or difficult to answer? |
|  | Is someone in your life keeping you from doing something you want to do related to avoiding pregnancy? | No, not at all 1  Yes, a little 2  Yes, somewhat (as in more than a little) 3  Yes, very well/very much 4  I don’t understand 5 | 1. **Please tell me why you selected that answer.** 2. **Was the question confusing or difficult to answer in any way?**    1. [IF YES]: What specifically made the question confusing or difficult to answer? 3. ***IF THEY DON’T UNDERSTAND, REPEAT THE QUESTION AND***What specifically made the question confusing or difficult to answer? |
|  | Is there anyone in your life who has too much influence over what you are doing or not doing to avoid pregnancy? | No, not at all 1  Yes, a little 2  Yes, somewhat (as in more than a little) 3  Yes, very well/very much 4  I don’t understand 5 | 1. **Please tell me why you selected that answer.** 2. **Was the question confusing or difficult to answer in any way?**    1. [IF YES]: What specifically made the question confusing or difficult to answer? 3. ***IF THEY DON’T UNDERSTAND, REPEAT THE QUESTION AND***What specifically made the question confusing or difficult to answer? |
|  | Do you worry about what others will think if you choose to use a method to avoid pregnancy? | No, not at all 1  Yes, a little 2  Yes, somewhat (as in more than a little) 3  Yes, very well/very much 4  I don’t understand 5 | 1. **Please tell me why you selected that answer.** 2. **Was the question confusing or difficult to answer in any way?**    1. [IF YES]: What specifically made the question confusing or difficult to answer? 3. ***IF THEY DON’T UNDERSTAND, REPEAT THE QUESTION AND***What specifically made the question confusing or difficult to answer? |
|  | Do you worry about what others will think if you choose not to use a method to avoid pregnancy? | No, not at all 1  Yes, a little 2  Yes, somewhat (as in more than a little) 3  Yes, very well/very much 4  I don’t understand 5 | 1. **Please tell me why you selected that answer.** 2. **Was the question confusing or difficult to answer in any way?**    1. [IF YES]: What specifically made the question confusing or difficult to answer? 3. ***IF THEY DON’T UNDERSTAND, REPEAT THE QUESTION AND***What specifically made the question confusing or difficult to answer? |
|  | Are you able to do the things you want to do when it comes to avoiding or not avoiding pregnancy without worrying that someone will hurt you? | No, not at all 1  Yes, a little 2  Yes, somewhat (as in more than a little) 3  Yes, very well/very much 4  I don’t understand 5 | 1. **Please tell me why you selected that answer.** 2. **Was the question confusing or difficult to answer in any way?**    1. [IF YES]: What specifically made the question confusing or difficult to answer? 3. ***IF THEY DON’T UNDERSTAND, REPEAT THE QUESTION AND***What specifically made the question confusing or difficult to answer? |
|  | Could you use methods to avoid pregnancy without your partner knowing if you want to? | No, not at all 1  Yes, a little 2  Yes, somewhat (as in more than a little) 3  Yes, very well/very much 4  I don’t understand 5 | 1. **Please tell me why you selected that answer.** 2. **Was the question confusing or difficult to answer in any way?**    1. [IF YES]: What specifically made the question confusing or difficult to answer? 3. ***IF THEY DON’T UNDERSTAND, REPEAT THE QUESTION AND***What specifically made the question confusing or difficult to answer? |
|  | Are you able to control who in your family knows whether you are doing something to avoid pregnancy? | No, not at all 1  Yes, a little 2  Yes, somewhat (as in more than a little) 3  Yes, very well/very much 4  I don’t understand 5 | 1. **Please tell me why you selected that answer.** 2. **Was the question confusing or difficult to answer in any way?**    1. [IF YES]: What specifically made the question confusing or difficult to answer? 3. ***IF THEY DON’T UNDERSTAND, REPEAT THE QUESTION AND***What specifically made the question confusing or difficult to answer? |
|  | Do you think you have the right to decide what to do related to avoiding pregnancy even if a religious leader—e.g., a priest/Imam/pastor-wants you to do something else? | No, not at all 1  Yes, a little 2  Yes, somewhat (as in more than a little) 3  Yes, very well/very much 4  I don’t understand 5 | 1. **Please tell me why you selected that answer.** 2. **Was the question confusing or difficult to answer in any way?**    1. [IF YES]: What specifically made the question confusing or difficult to answer? 3. ***IF THEY DON’T UNDERSTAND, REPEAT THE QUESTION AND***What specifically made the question confusing or difficult to answer? |
|  | Do you believe you have the right to decide what to do related to avoiding pregnancy even if a community leader or elder wants you to do something else? | No, not at all 1  Yes, a little 2  Yes, somewhat (as in more than a little) 3  Yes, very well/very much 4  I don’t understand 5 | 1. **Please tell me why you selected that answer.** 2. **Was the question confusing or difficult to answer in any way?**    1. [IF YES]: What specifically made the question confusing or difficult to answer? 3. ***IF THEY DON’T UNDERSTAND, REPEAT THE QUESTION AND***What specifically made the question confusing or difficult to answer? |
|  | In the area of avoiding pregnancy, do you have the right to decide what to to do even if people in your community want you to do something else? | No, not at all 1  Yes, a little 2  Yes, somewhat (as in more than a little) 3  Yes, very well/very much 4  I don’t understand 5 | 1. **Please tell me why you selected that answer.** 2. **Was the question confusing or difficult to answer in any way?**    1. [IF YES]: What specifically made the question confusing or difficult to answer? 3. ***IF THEY DON’T UNDERSTAND, REPEAT THE QUESTION AND***What specifically made the question confusing or difficult to answer? |

**Questionnaire II: #33-64 (ODK will randomly select respondent for Questionnaire I or Questionnaire II)**

**Instructions:** Read aloud all four response options “No, not at all; Yes, a little; Yes, somewhat; Yes, very well/very much”.

| **#** | **Item** | | **Response Option** | **Questions/Probes** | |
| --- | --- | --- | --- | --- | --- |
| **Domain 1** | | | | | |
|  | | Do you think it is ok for a healthcare provider to refuse to take out a pregnancy prevention method that a person wants to take out? | No, not at all 1  Yes, a little 2  Yes, somewhat (as in more than a little) 3  Yes, very well/very much 4  I don’t understand 5 | | 1. **Please tell me why you selected that answer.** 2. **Was the question confusing or difficult to answer in any way?**    1. [IF YES]: What specifically made the question confusing or difficult to answer? 3. ***IF THEY DON’T UNDERSTAND, REPEAT THE QUESTION AND***What specifically made the question confusing or difficult to answer? |
|  | | Do you think it is ok for a healthcare provider to hide from you about ways to avoid pregnancy, that is, making sure it does not happen when you do not want it to? | No, not at all 1  Yes, a little 2  Yes, somewhat (as in more than a little) 3  Yes, very well/very much 4  I don’t understand 5 | | 1. **Please tell me why you selected that answer.** 2. **Was the question confusing or difficult to answer in any way?**    1. [IF YES]: What specifically made the question confusing or difficult to answer? 3. ***IF THEY DON’T UNDERSTAND, REPEAT THE QUESTION AND***What specifically made the question confusing or difficult to answer? |
|  | | Do you believe that people should have the right to be informed of their options for avoiding pregnancy? | No, not at all 1  Yes, a little 2  Yes, somewhat (as in more than a little) 3  Yes, very well/very much 4  I don’t understand 5 | | 1. **Please tell me why you selected that answer.** 2. **Was the question confusing or difficult to answer in any way?**    1. [IF YES]: What specifically made the question confusing or difficult to answer? 3. ***IF THEY DON’T UNDERSTAND, REPEAT THE QUESTION AND***What specifically made the question confusing or difficult to answer? |
|  | | Do you think people should have the right to choose from a range of methods to avoid pregnancy if they want to use one? | No, not at all 1  Yes, a little 2  Yes, somewhat (as in more than a little) 3  Yes, very well/very much 4  I don’t understand 5 | | 1. **Please tell me why you selected that answer.** 2. **Was the question confusing or difficult to answer in any way?**    1. [IF YES]: What specifically made the question confusing or difficult to answer? 3. ***IF THEY DON’T UNDERSTAND, REPEAT THE QUESTION AND***What specifically made the question confusing or difficult to answer? |
|  | | Should people have the right to switch to a different method to avoid pregnancy if they do not like their current one? | No, not at all 1  Yes, a little 2  Yes, somewhat (as in more than a little) 3  Yes, very well/very much 4  I don’t understand 5 | | 1. **Please tell me why you selected that answer.** 2. **Was the question confusing or difficult to answer in any way?**    1. [IF YES]: What specifically made the question confusing or difficult to answer? 3. ***IF THEY DON’T UNDERSTAND, REPEAT THE QUESTION AND***What specifically made the question confusing or difficult to answer? |
|  | | Do you think young people are often denied a method to avoid pregnancy due to people’s beliefs about whether they should be having sex? | No, not at all 1  Yes, a little 2  Yes, somewhat (as in more than a little) 3  Yes, very well/very much 4  I don’t understand 5 | | 1. **Please tell me why you selected that answer.** 2. **Was the question confusing or difficult to answer in any way?**    1. [IF YES]: What specifically made the question confusing or difficult to answer? 3. ***IF THEY DON’T UNDERSTAND, REPEAT THE QUESTION AND***What specifically made the question confusing or difficult to answer? |
|  | | Do you think how people view women where you live, affects women’s choices for avoiding pregnancy? | No, not at all 1  Yes, a little 2  Yes, somewhat (as in more than a little) 3  Yes, very well/very much 4  I don’t understand 5 | | 1. **Please tell me why you selected that answer.** 2. **Was the question confusing or difficult to answer in any way?**    1. [IF YES]: What specifically made the question confusing or difficult to answer? 3. ***IF THEY DON’T UNDERSTAND, REPEAT THE QUESTION AND***What specifically made the question confusing or difficult to answer? |
|  | | Do people’s expectations for men where you live influence whether men use methods to avoid pregnancy? | No, not at all 1  Yes, a little 2  Yes, somewhat (as in more than a little) 3  Yes, very well/very much 4  I don’t understand 5 | | 1. **Please tell me why you selected that answer.** 2. **Was the question confusing or difficult to answer in any way?**    1. [IF YES]: What specifically made the question confusing or difficult to answer? 3. ***IF THEY DON’T UNDERSTAND, REPEAT THE QUESTION AND***What specifically made the question confusing or difficult to answer? |
|  | | Do you think how people view unmarried women where you live affects unmarried women’s choices for avoiding pregnancy? | No, not at all 1  Yes, a little 2  Yes, somewhat (as in more than a little) 3  Yes, very well/very much 4  I don’t understand 5 | | 1. **Please tell me why you selected that answer.** 2. **Was the question confusing or difficult to answer in any way?**    1. [IF YES]: What specifically made the question confusing or difficult to answer? 3. ***IF THEY DON’T UNDERSTAND, REPEAT THE QUESTION AND***What specifically made the question confusing or difficult to answer? |
|  | | Do you think it is difficult for women to tell a partner they disagree with them about avoiding pregnancy? | No, not at all 1  Yes, a little 2  Yes, somewhat (as in more than a little) 3  Yes, very well/very much 4  I don’t understand 5 | | 1. **Please tell me why you selected that answer.** 2. **Was the question confusing or difficult to answer in any way?**    1. [IF YES]: What specifically made the question confusing or difficult to answer? 3. ***IF THEY DON’T UNDERSTAND, REPEAT THE QUESTION AND***What specifically made the question confusing or difficult to answer? |
|  | | Do you have the final say on whether or not you use a method to avoid pregnancy? | No, not at all 1  Yes, a little 2  Yes, somewhat (as in more than a little) 3  Yes, very well/very much 4  I don’t understand 5 | | 1. **Please tell me why you selected that answer.** 2. **Was the question confusing or difficult to answer in any way?**    1. [IF YES]: What specifically made the question confusing or difficult to answer? 3. ***IF THEY DON’T UNDERSTAND, REPEAT THE QUESTION AND***What specifically made the question confusing or difficult to answer? |
|  | | Do you have the power to control if you use a method to avoid pregnancy? | No, not at all 1  Yes, a little 2  Yes, somewhat (as in more than a little) 3  Yes, very well/very much 4  I don’t understand 5 | | 1. **Please tell me why you selected that answer.** 2. **Was the question confusing or difficult to answer in any way?**    1. [IF YES]: What specifically made the question confusing or difficult to answer? 3. ***IF THEY DON’T UNDERSTAND, REPEAT THE QUESTION AND***What specifically made the question confusing or difficult to answer? |
|  | | Do you decide for yourself who is involved in your decisions about avoiding pregnancy? | No, not at all 1  Yes, a little 2  Yes, somewhat (as in more than a little) 3  Yes, very well/very much 4  I don’t understand 5 | | 1. **Please tell me why you selected that answer.** 2. **Was the question confusing or difficult to answer in any way?**    1. [IF YES]: What specifically made the question confusing or difficult to answer? 3. ***IF THEY DON’T UNDERSTAND, REPEAT THE QUESTION AND***What specifically made the question confusing or difficult to answer? |
|  | | Is it your decision who you tell or do not tell about if you are doing something to avoid pregnancy? | No, not at all 1  Yes, a little 2  Yes, somewhat (as in more than a little) 3  Yes, very well/very much 4  I don’t understand 5 | | 1. **Please tell me why you selected that answer.** 2. **Was the question confusing or difficult to answer in any way?**    1. [IF YES]: What specifically made the question confusing or difficult to answer? 3. ***IF THEY DON’T UNDERSTAND, REPEAT THE QUESTION AND***What specifically made the question confusing or difficult to answer? |
|  | | Do you decide for yourself whose opinions you listen to about avoiding pregnancy? | No, not at all 1  Yes, a little 2  Yes, somewhat (as in more than a little) 3  Yes, very well/very much 4  I don’t understand 5 | | 1. **Please tell me why you selected that answer.** 2. **Was the question confusing or difficult to answer in any way?**    1. [IF YES]: What specifically made the question confusing or difficult to answer? 3. ***IF THEY DON’T UNDERSTAND, REPEAT THE QUESTION AND***What specifically made the question confusing or difficult to answer? |
|  | | Are you confident that you can make a choice about whether or not to use a method to avoid pregnancy? | No, not at all 1  Yes, a little 2  Yes, somewhat (as in more than a little) 3  Yes, very well/very much 4  I don’t understand 5 | | 1. **Please tell me why you selected that answer.** 2. **Was the question confusing or difficult to answer in any way?**    1. [IF YES]: What specifically made the question confusing or difficult to answer? 3. ***IF THEY DON’T UNDERSTAND, REPEAT THE QUESTION AND***What specifically made the question confusing or difficult to answer? |
|  | | Do you know who to talk to if you need information or support related to avoiding pregnancy? | No, not at all 1  Yes, a little 2  Yes, somewhat (as in more than a little) 3  Yes, very well/very much 4  I don’t understand 5 | | 1. **Please tell me why you selected that answer.** 2. **Was the question confusing or difficult to answer in any way?**    1. [IF YES]: What specifically made the question confusing or difficult to answer? 3. ***IF THEY DON’T UNDERSTAND, REPEAT THE QUESTION AND***What specifically made the question confusing or difficult to answer? |
|  | | If a partner or other family member did not support your choice, would you still choose to do what is best for you related to avoiding pregnancy? | No, not at all 1  Yes, a little 2  Yes, somewhat (as in more than a little) 3  Yes, very well/very much 4  I don’t understand 5 | | 1. **Please tell me why you selected that answer.** 2. **Was the question confusing or difficult to answer in any way?**    1. [IF YES]: What specifically made the question confusing or difficult to answer? 3. ***IF THEY DON’T UNDERSTAND, REPEAT THE QUESTION AND***What specifically made the question confusing or difficult to answer? |
|  | | Are you confident that you could use a method to avoid pregnancy even if someone else, like a partner or other family member did not want you to? | No, not at all 1  Yes, a little 2  Yes, somewhat (as in more than a little) 3  Yes, very well/very much 4  I don’t understand 5 | | 1. **Please tell me why you selected that answer.** 2. **Was the question confusing or difficult to answer in any way?**    1. [IF YES]: What specifically made the question confusing or difficult to answer? 3. ***IF THEY DON’T UNDERSTAND, REPEAT THE QUESTION AND***What specifically made the question confusing or difficult to answer? |
|  | | Are you confident that you could refuse to use a method to avoid pregnancy even if someone else, like a partner or other family member, wanted you to use one? | No, not at all 1  Yes, a little 2  Yes, somewhat (as in more than a little) 3  Yes, very well/very much 4  I don’t understand 5 | | 1. **Please tell me why you selected that answer.** 2. **Was the question confusing or difficult to answer in any way?**    1. [IF YES]: What specifically made the question confusing or difficult to answer? 3. ***IF THEY DON’T UNDERSTAND, REPEAT THE QUESTION AND***What specifically made the question confusing or difficult to answer? |
| **Domain 2** | | | | | |
|  | | Read aloud, “Yes or no” followed by asking “No or strong no” OR “Yes or strong yes”. | | |  |
|  | | Are you free to use a method to avoid pregnancy if you want to? | Strongly no 1  No 2  Yes 3  Strongly yes 4  I don’t understand 5 | | 1. **Please tell me why you selected that answer.** 2. **Was the question confusing or difficult to answer in any way?**    1. [IF YES]: What specifically made the question confusing or difficult to answer? 3. ***IF THEY DON’T UNDERSTAND, REPEAT THE QUESTION AND***What specifically made the question confusing or difficult to answer? |
|  | | Are you free not to use or to stop using a method to avoid pregnancy if you want to? | Strongly no 1  No 2  Yes 3  Strongly yes 4  I don’t understand 5 | | 1. **Please tell me why you selected that answer.** 2. **Was the question confusing or difficult to answer in any way?**    1. [IF YES]: What specifically made the question confusing or difficult to answer? 3. ***IF THEY DON’T UNDERSTAND, REPEAT THE QUESTION AND***What specifically made the question confusing or difficult to answer? |
|  | | Would you be able to use a method to avoid pregnancy if you wanted to? | Strongly no 1  No 2  Yes 3  Strongly yes 4  I don’t understand 5 | | 1. **Please tell me why you selected that answer.** 2. **Was the question confusing or difficult to answer in any way?**    1. [IF YES]: What specifically made the question confusing or difficult to answer? 3. ***IF THEY DON’T UNDERSTAND, REPEAT THE QUESTION AND***What specifically made the question confusing or difficult to answer? |
|  | | Would you be able to stop using or to not use method to avoid pregnancy if you wanted to? | Strongly no 1  No 2  Yes 3  Strongly yes 4  I don’t understand 5 | | 1. **Please tell me why you selected that answer.** 2. **Was the question confusing or difficult to answer in any way?**    1. [IF YES]: What specifically made the question confusing or difficult to answer? 3. ***IF THEY DON’T UNDERSTAND, REPEAT THE QUESTION AND***What specifically made the question confusing or difficult to answer? |
|  | | Could you get the method of your choice if you want to use a method to avoid pregnancy? | Strongly no 1  No 2  Yes 3  Strongly yes 4  I don’t understand 5 | | 1. **Please tell me why you selected that answer.** 2. **Was the question confusing or difficult to answer in any way?**    1. [IF YES]: What specifically made the question confusing or difficult to answer? 3. ***IF THEY DON’T UNDERSTAND, REPEAT THE QUESTION AND***What specifically made the question confusing or difficult to answer? |
|  | | At this point in your life, do you have the information you want about avoiding pregnancy? | Strongly no 1  No 2  Yes 3  Strongly yes 4  I don’t understand 5 | | 1. **Please tell me why you selected that answer.** 2. **Was the question confusing or difficult to answer in any way?**    1. [IF YES]: What specifically made the question confusing or difficult to answer? 3. ***IF THEY DON’T UNDERSTAND, REPEAT THE QUESTION AND***What specifically made the question confusing or difficult to answer? |
|  | | Is someone in your life making you do something you don’t want to do related to avoiding pregnancy? | Strongly no 1  No 2  Yes 3  Strongly yes 4  I don’t understand 5 | | 1. **Please tell me why you selected that answer.** 2. **Was the question confusing or difficult to answer in any way?**    1. [IF YES]: What specifically made the question confusing or difficult to answer? 3. ***IF THEY DON’T UNDERSTAND, REPEAT THE QUESTION AND***What specifically made the question confusing or difficult to answer? |
|  | | Could you talk to a pharmacist or healthcare worker about getting a method to avoid pregnancy if you wanted to? | Strongly no 1  No 2  Yes 3  Strongly yes 4  I don’t understand 5 | | 1. **Please tell me why you selected that answer.** 2. **Was the question confusing or difficult to answer in any way?**    1. [IF YES]: What specifically made the question confusing or difficult to answer? 3. ***IF THEY DON’T UNDERSTAND, REPEAT THE QUESTION AND***What specifically made the question confusing or difficult to answer? |
|  | | Do you have access to the money that you need to get a method to avoid pregnancy if you need to? | Strongly no 1  No 2  Yes 3  Strongly yes 4  I don’t understand 5 | | 1. **Please tell me why you selected that answer.** 2. **Was the question confusing or difficult to answer in any way?**    1. [IF YES]: What specifically made the question confusing or difficult to answer? 3. ***IF THEY DON’T UNDERSTAND, REPEAT THE QUESTION AND***What specifically made the question confusing or difficult to answer? |
|  | | If you want to use a method to avoid pregnancy, do you know where to get methods you can afford? | Strongly no 1  No 2  Yes 3  Strongly yes 4  I don’t understand 5 | | 1. **Please tell me why you selected that answer.** 2. **Was the question confusing or difficult to answer in any way?**    1. [IF YES]: What specifically made the question confusing or difficult to answer? 3. ***IF THEY DON’T UNDERSTAND, REPEAT THE QUESTION AND***What specifically made the question confusing or difficult to answer? |
|  | | If you want to use a method to avoid pregnancy, do you know where to get the method you want? | No, not at all 1  Yes, a little 2  Yes, somewhat (as in more than a little) 3  Yes, very well/very much 4  I don’t understand 5 | | 1. **Please tell me why you selected that answer.** 2. **Was the question confusing or difficult to answer in any way?**    1. [IF YES]: What specifically made the question confusing or difficult to answer? 3. ***IF THEY DON’T UNDERSTAND, REPEAT THE QUESTION AND***What specifically made the question confusing or difficult to answer? |
|  | | If you needed help related to avoiding pregnancy, could you find transportation to a healthcare facility for help? | Strongly no 1  No 2  Yes 3  Strongly yes 4  I don’t understand 5 | | 1. **Please tell me why you selected that answer.** 2. **Was the question confusing or difficult to answer in any way?**    1. [IF YES]: What specifically made the question confusing or difficult to answer? 3. ***IF THEY DON’T UNDERSTAND, REPEAT THE QUESTION AND***What specifically made the question confusing or difficult to answer? |
|  | | If you wanted to use a new method to avoid pregnancy could you figure out how to get one? | Strongly no 1  No 2  Yes 3  Strongly yes 4  I don’t understand 5 | | 1. **Please tell me why you selected that answer.** 2. **Was the question confusing or difficult to answer in any way?**    1. [IF YES]: What specifically made the question confusing or difficult to answer? 3. ***IF THEY DON’T UNDERSTAND, REPEAT THE QUESTION AND***What specifically made the question confusing or difficult to answer? |
|  | | If something or someone stood in your way, could you find a way to use a method to avoid pregnancy? | Strongly no 1  No 2  Yes 3  Strongly yes 4  I don’t understand 5 | | 1. **Please tell me why you selected that answer.** 2. **Was the question confusing or difficult to answer in any way?**    1. [IF YES]: What specifically made the question confusing or difficult to answer? 3. ***IF THEY DON’T UNDERSTAND, REPEAT THE QUESTION AND***What specifically made the question confusing or difficult to answer? |
