# Supplementary material for: Development and Validation of the Agency in Contraceptive Decisions Scale in Uganda and Nigeria
Source: Stud Fam Plann. 2025 Sep 22;56(3):655–75. doi: 10.1111/sifp.70033 (PMC12501711; doi:10.1111/sifp.70033)
Supplement: Supplementary file 2 — Appedix 2 [file SIFP-56-655-s002.docx]

**Appendix Table 1. Item pool candidates not included in the final Agency in Contraceptive Decisions Scale, Nigeria and Uganda (N=3,002)**

| **Items** | **Mean(SD)** |
| --- | --- |
| Do you feel certain about whether you want to do anything to avoid pregnancy? | 2.7(0.6) |
| If you want to use a method to avoid pregnancy, do you know where to get it? | 2.8(0.5) |
| Do you know who to talk to if you need information or support related to avoiding pregnancy? | 2.7(0.6) |
| Do you believe it is your right to decide whether to use (or not) a method to avoid pregnancy? | 2.7(0.5) |
| Do you believe **married women** should be able to use a method to avoid pregnancy when they want to? | 2.7(0.5) |
| Do you believe **unmarried women** should be able to use a method to avoid pregnancy when they want to? | 2.3(1.0) |
| Do you believe you have the right to refuse a healthcare provider who tries to make you use a method to avoid pregnancy? | 2.7(0.6) |
| Should women be able to use methods to avoid pregnancy without their partners knowing if that’s what they want? | 1.7(1.3) |
| Is it ever ok for a healthcare provider to refuse to take out a method to avoid pregnancy, say an IUD or implant, that a person wants taken out? | 2.7(0.7) |
| Do you have the right to decide what to do related to avoiding pregnancy even if people in your community want you to do something else? | 2.8(0.5) |
| Do you have the right to decide what to do related to avoiding pregnancy even if elders want you do so something else? | 2.7(0.6) |
| Do you believe that unmarried women’s choices for avoiding pregnancy are unfairly limited? | 1.7(1.2) |
| Do you think women are often unable to discuss what they want related to avoiding pregnancy with their partners? | 2.2(1.0) |
| Do you believe that women have fewer chances to be successful in life? | 2.2(1.1) |
| Do you believe that we would have fewer problems if we treated people more equally? | 2.7(0.6) |
| Could you get a method to avoid pregnancy without people in your community knowing if you wanted to? | 2.6(0.8) |
| Do you worry about what others will think if you choose to use a method to avoid pregnancy? | 2.5(0.9) |
| Now I would like to ask you the opposite, do you worry about what others will think if you choose NOT to use a method to avoid pregnancy? | 2.0(1.2) |
| Could you use methods to avoid pregnancy without your partner knowing if you want to? | 1.9(1.3) |
| If someone pressured you to use a method to avoid pregnancy, could you refuse if you did not want to use it? | 2.7(0.6) |
| Are you confident that you can make a choice about whether or not to use a method to avoid pregnancy? | 2.7(0.5) |
| Imagine that you wanted to use a method to avoid pregnancy…Would you be able to use one? | 2.7(0.5) |
| Now I would like to ask you the opposite, imagine that you did not want to use a method to avoid pregnancy…Would you be able to *not* use one? | 2.6(0.7) |
| Are you free to do what you want related to avoiding pregnancy? | 2.7(0.6) |
| Does lack of money make it difficult for you to do what you want related to avoiding pregnancy? | 2.2(1.1) |
| Do you feel confident about what you are doing or not doing to avoid pregnancy? | 2.7(0.5) |
| Can you control who in your family knows whether you are doing something to avoid pregnancy? | 2.6(0.8) |
